# The continuous flow synthesis of azos

**DOI:** 10.1007/s41981-024-00307-2

**Published:** 2024-01-26

**Authors:** Adam T. McCormack, John C. Stephens

**Affiliations:** 1 Department of Chemistry, Maynooth University, Maynooth, Ireland; 2 The Kathleen Lonsdale Institute for Human Health Research, Maynooth University, Maynooth, Ireland

**Keywords:** Continuous flow synthesis, Azo, Dye, Diazonium, Microreactor

## Abstract

Azo compounds find use in many areas of science, displaying crucial properties for important applications as photoconductive organic pigments, fluorescent quenchers, paints, cosmetics, inks, and in the large and valuable dye industry. Due to the unstable intermediates, and the exothermic and fast reactions used in their synthesis, high value azo compounds are excellent candidates for continuous flow manufacturing. This comprehensive review covers the progress made to date on developing continuous flow systems for azo synthesis and reflects on the main challenges still to be addressed, including scale up, conversion, product purity, and environmental impact. The further development of integrated continuous flow processes has the potential to help tackle these challenges and deliver improved methods for azo compound generation.

## Introduction

Azos are valuable compounds with many important uses including application as photoconductive organic pigments [1], fluorescent quenchers [2, 3], paints [4], cosmetics [5], inks and dyes [6], and in colour photography [7]. Azos contain the characteristic functional group R-N=N-R’, where typically the R and R’ groups are aryl. Aromatic azo compounds tend to be coloured due to their conjugated molecular structure and absorb light in the visible spectrum (400-700 nm). As a result, azo compounds have found use as synthetic dyes, and are the category of dye produced in the largest quantities (over 60%) [8, 9]. Azo dyes offer some advantages over other synthetic dyes and can, in some cases, employ inexpensive raw materials, short industrial process, and relatively straightforward synthetic methods [10]. Azo dyes have their drawbacks, and consideration must be given to the damaging impact of azo dye by-products and waste streams on the environment [7, 8]. Azo compounds are not only valuable compounds in the chemical industries but also find many important uses in medicinal and biomedical fields, where they are utilised as prodrugs [9–12], drug-coating materials [13], and antimicrobial agents [14]. Azo compounds also serve as photo switches and are the subject of intense research with the discovery that their photophysics’ properties can be tuned by the introduction of different substituents to the aryls/heteroaryls. Some of the most important of these properties include the control of the positions of absorptions bands [15].

Azo compounds can be synthesized using several approaches including azo coupling, the most common method reported, Mills reactions and Wallach reactions [13, 14, 16]. In the dye industry, azos are often prepared in batch reactors using azo coupling reactions and take advantage of the simple, low cost nature of the equipment involved [10]. However, batch processing can suffer from low yields, non-uniformity of reactant mixing, different chromatic light properties between different batches, the use of exothermic diazotization and azo coupling reactions, and the potentially hazardous isolation of unstable diazonium salts [10]. As such, there has been a recent increase in interest in the use of continuous flow synthesis for the generation of azo compounds due to its potential to solve some of the issues found with azo batch processes. The use of smaller continuous flow reactors over batch reactors offers several possible benefits including enhanced heat-mass transfer, reproducibility, scale up, and improved safety profile [17–19]. Exothermic processes, such as azo formation, can be problematic when employing batch technologies and the enhanced heat-mass transfer of continuous flow reactors can significantly improve these processes. Additionally, the generation of large quantities of unstable intermediates, such as diazo species, can be avoided through the use of smaller continuous flow reactors yet still allow the generation of large quantities of final product. The continuous flow synthesis of azo compounds has been reported in both microfluidic and larger flow systems and this publication reviews advances made in the continuous flow synthesis of azo compounds.

## Azo coupling reaction

**Fig. 1:**
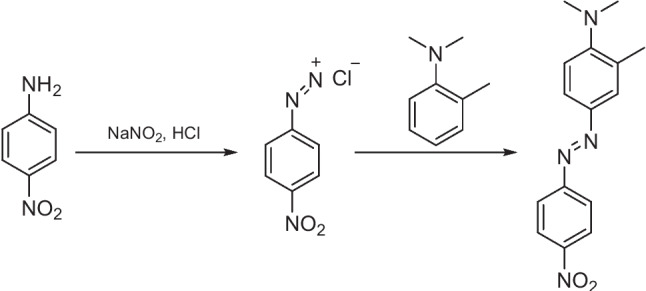
Reaction scheme of diazotization and azo coupling reaction, respectively

The synthesis of azo compounds is most often performed through a two-step process: diazotisation of an aromatic primary amine at low temperatures and then reaction with an electron rich aromatic nucleophile in the azo coupling step (Fig. 1). Diazonium salts are weak electrophiles but will react with electron rich species, including arenes with electron donor substituents (e.g. amine or hydroxyl), to give azo products [15, 20]. Typically, the azo group is formed *para* to the electron donor substituent on the nucleophilic arene but can form at the *ortho* position when the *para* position is already occupied. The azo coupling reaction is an exothermic reaction and can release substantial amounts of heat [21]. An azo coupling rection often needs to be carried out with both fast agitation and slow addition of the diazonium salt solution, as this prevents local overheating as well as unwanted by-products [21]. Azo coupling reactions can have many issues, such as high energy consumption, lengthy reaction times, and relatively low production efficiencies, which can be observed with some batch processes [21]. In such azo batch processes, ineffective heat and mass transfer can cause variation in the local conditions at different locations in the reactor, such as local temperature, local pH, and local reactant concentrations, and lead to poor results and variation in colour [21]. An advantage of continuous flow technology in the synthesis of azo products, and synthesis in general, is the higher degree of reaction control and ability to more precisely control reaction parameters and conditions [22]. Additionally, diazotization and azo coupling reactions are exothermic and diazonium salt intermediates are known to be hazardous due to their potential explosive and unstable nature [23]. Generally, within industry, azo pigments are often synthesized by batch processes using stirred tasks with volumes ranging between 20 m^3^ and 80 m^3^. These azo products synthesized in batch processes are prone to broad particle-size distribution, with a large particle size and a lower conversion rate of the azo coupling reaction leading to poorer colour properties [24]

## Continuous flow synthesis of azos: 2002-2009

**Fig. 2:**
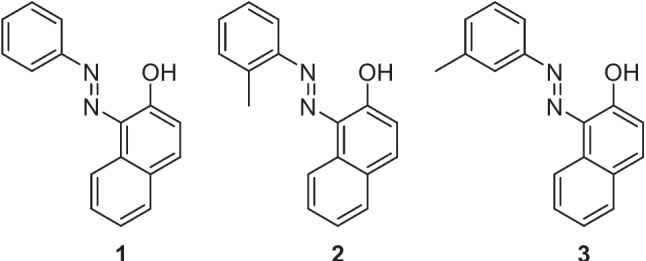
Azo dye products reported by Wootton et al. [25]

**Fig. 3:**
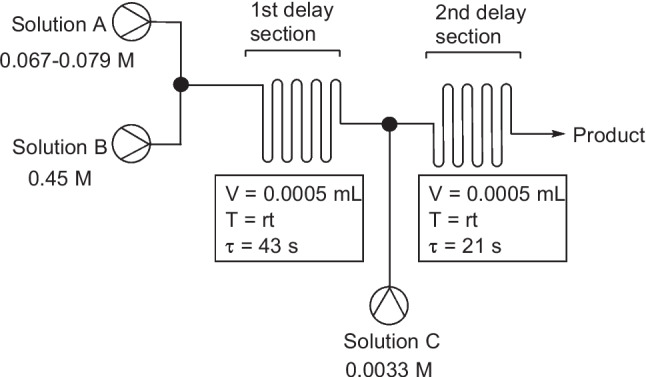
Continuous flow synthesis of azos as described by Wootten et al [25]. Solution A (0.067-0.079 M) corresponding aniline, conc. hydrochloric acid, water:DMF (1:6); solution B (0.45 M) sodium nitrite, water:DMF (1:5); solution C (0.0033 M) β-naphthol, sodium hydroxide solution, water:DMF (1:14.5). Solutions A and B had individual flow rates of 3.5 μl min^-1^. Solution C had a flow rate of 7 μl min^-1^

The first report of a continuous flow system for the synthesis of azo molecules was by Wootton et al*.* in 2002 [25]. Therein, they described the generation of three azo dyes (Fig. 2) using nanoreactors in a continuous flow multistage process. The nanoreactor set up (Wootten denoted nanoreactors as reactors with an instantaneous reaction volume most conveniently measured in nanolitres) consisted of two Y junctions separated by serpentine delay section. A second delay section preceded the exhaust, at which the samples were collected for analysis, see Fig. 3 for experiment details. The authors reported that the mixing of the reagents relied on diffusion and that azo dye formation was evidenced by the immediate formation of a persistent red colour in the efferent streams of the second delay section. Yields were not determined, but IR spectroscopy was used to confirm conversion, although this conversion varied considerably for the three azos generated. Compound 1 gave a 52 % conversion, compound 2 a 23 % conversion, and compound 3 a 9 % conversion. Wootton et al*.* stated that reaction optimisation was not performed for the diazotisation step and that this was most likely responsible for variation in conversion and suggested that optimisation of this step could have led to improved yields. The authors concluded that the safe nature of the procedure as described showed clear advantages over bulk scale syntheses and suggested that with optimisation for diazonium formation it may offer an alternative to existing industrial routes.

**Fig. 4:**
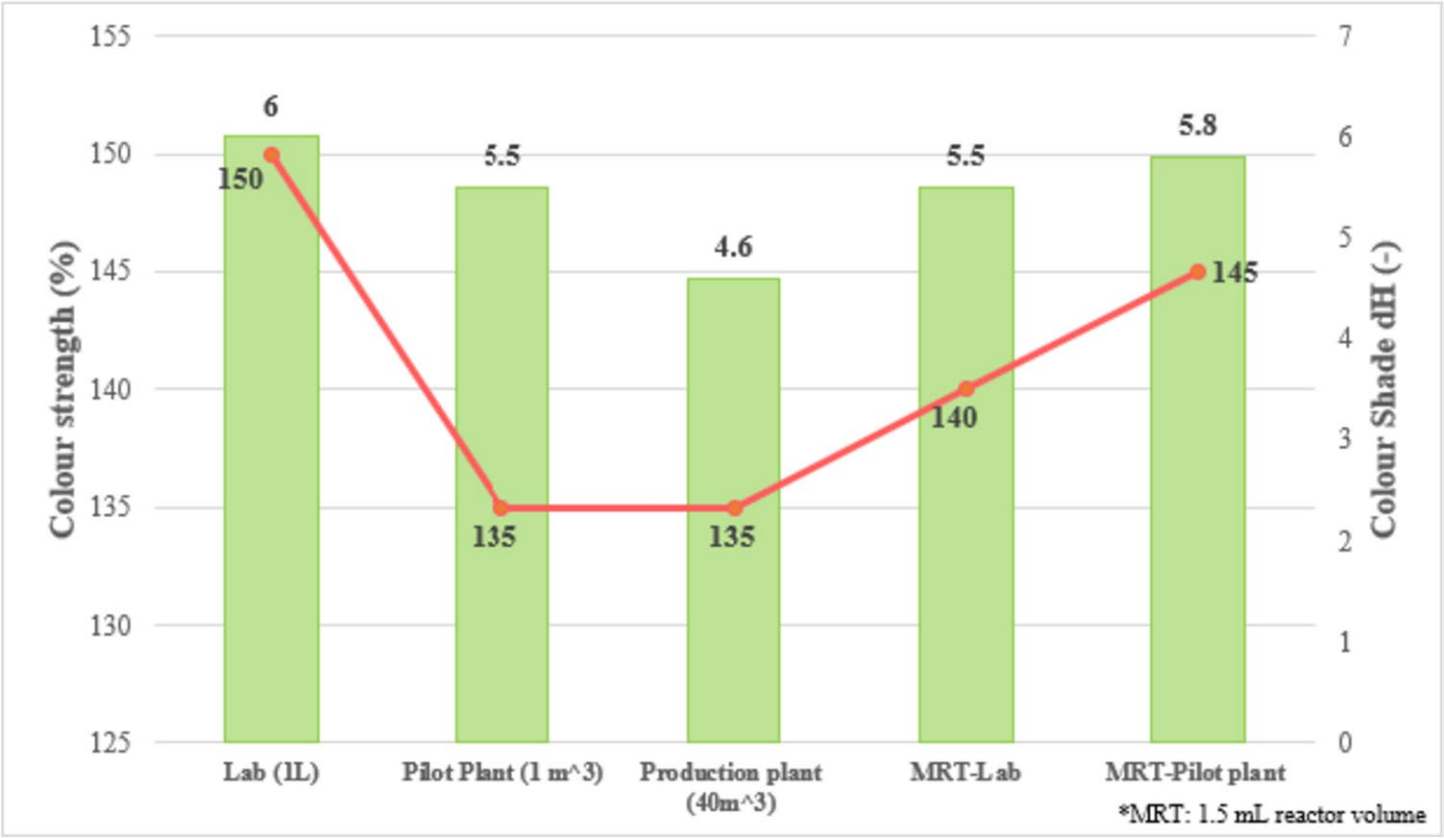
Pigment quality: scaling-up (batch synthesis) vs. numbering-up of CPC-microreactors for small-scale production of a yellow azo pigment. Adapted from source [26]

In 2004, Wille at al. reported a study that compared batch scale-up with the numbering up of flow systems (microreaction technology, MRT) in the synthesis of azo dyes (structures not reported) [26]. The term numbering up refers to operating several replicate MRT systems and not increasing the scale of a single flow system. Three scales were used for the batch synthesis of a yellow azo dye: lab scale (1 L), pilot scale (1 m^3^), and production scale (40 m^3^). Here, results for colour shade (describes nuances of a deviation from the target colour, e.g. yellow with a slight green nuance at the larger scale) and colour strength (pigment quality, how strongly coloured the dye is, given in percent) showed a significant drop off as scale increased, Fig. 4. It was suggested that this is primarily due to increased reaction volume and the loss of mixing quality when moving the batch process from lab scale to pilot scale/full production. In contrast, reaction and mixing volumes did not change from MRT-lab to MRT-pilot plant scale (due to the numbering up approach) and, therefore, pigment quality of MRT-lab scale was reproducible in the MRT-pilot plant scale, Fig. 4. However, the output of one pilot plant batch operation was reported to be about 80 kg per batch operation and hour, while the output for the numbered up flow system was at a significantly lower scale of 1 kg/h. For the MRT process, a solution of the diazonium salt and of the coupling component were separately and continuously fed into the CPC (Cellular Process Chemistry Systems GmbH) microreactor using multiple piston pumps. Coupling of both the yellow and red model pigments (structures not reported) were performed at room temperature and at set pH values of 6.3 for the yellow pigment and 5.0 for the red pigment (pH-values were adjusted by use of an additional pump for dosing of an alkaline solution as buffer).

The same report also looked at the performance of four microdevices/mixers that were commercially available at that time. The microdevices/mixers investigated were a conventional 0.5 in. helical static mixer, a standard caterpillar mixer of IMM, a Micro-Jet reactor, which was latter equipped with two coaxial nozzles (∅ = 400 μm), and a PCR-microreactor with an integrated heat ex-changer. To do this mixer comparison, Wille et al. measured the absorption of formed iodine at λ = 352 nm generated by the continuous mixing of 30 kg/h NaAc–KI–KIO_3_-solution with 30 kg/h HCl-solution (Villermaux’s reaction [27]) where higher detected iodine concentration indicated less effective mixing. The experiment reveals that the best mixing properties were found with the Micro-Jet reactor, where two mass transfer accelerating effects coincide: (i) mixing in small volumes and (ii) maximum transformation of kinetic energy of two fluid beams into mixing energy (the fluid beams meet each other in a collision point in the centre of a mixing zone). However, a major limitation was reported in that a maximum flow rate ratio of 1:1.5 should not be exceeded otherwise the beam with higher flow rate and, thus stronger momentum, is too powerful with respect to the weaker fluid beam emerging from the opposite nozzle. While Wille and co-workers saw the potential of flow systems for synthesis of azo dyes they also noted that the choice of mixing device was very important and that at that time the quality of microdevices available needed to be improved.

**Fig. 5:**
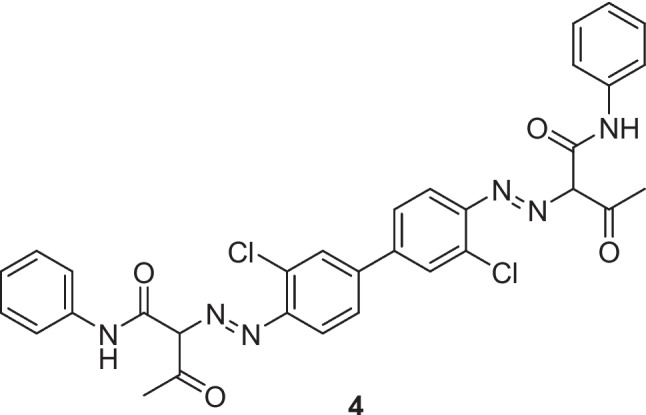
Azo dye Yellow 12 produced by Penneman et al [28]

In 2005, Penneman et al*.* reported the generation of azo dye Yellow 12 particles (compound 4) in a semi-continuous process using an interdigital micromixer, Fig. 5 [28]. The experimental setup consisted of an interdigital micromixer (width of the microchannels of the mixing unit, 25 or 40 μm) fed by two HPLC pumps (Gilson-Abimed 303 with a preparative pump head SC100), a manometric module (Gilson 803C), and suction hoses with frits. The starting compounds were introduced as separate homogeneous liquid solutions (diazo component: 0.12 M diazotised 3,3′-dichlorobenzidine in hydrochloric acid; coupling component: 0.24 M in an aqueous sodium hydroxide solution). Flow rates of 10-, 30-, and 50-mL min^-1^ were employed in different runs. The first 10 mL of reaction mixture was discarded, and the resulting azo pigment suspension was collected and worked up, including heating to 80 °C to break up any aggregated pigment. A subsequent series of cooling, filtration, washing (with water), and drying steps ultimately led to the final pigment material. The resultant azo pigments were characterised by their (i) particle size distribution, (ii) rheological properties, and (iii) colour properties, namely the glossiness, transparency, and tinctorial power.

The authors stated that the flow process resulted in a smaller particle size, and in connection with that reduction the colour properties of the pigments were improved, i.e. the glossiness had increased by 73% and the transparency by 66% compared to the Yellow 12 standard [28]. In terms of flow rates effects, the rheological properties showed no clear change with increasing flow rate, however, the colour properties were improved with higher flow rates and it was suggested that this was due to better mixing efficiency in the micromixer at the higher flow rates.

In 2008, Kockmann et al*.* reported the use of a continuous flow process and T-shaped micromixers for the azo coupling step in the synthesis of pigment CLA1433 [29]. The research hypothesis was that enhanced conversion to CLA1433 would result when using T-shaped micromixers due to the rapid mixing that would occur. Separate diazonium and coupler solutions were prepared, with the diazonium solution prepared according to the process regulations for batch operation, but with a dilution factor of 10 with water to reduce the particle concentration in the product flow. The two solutions were mixed within the T-shaped convective mixer. Three T-shaped micromixers were used with symmetric inlet flow conditions and a mixing channel with rectangular cross section (300 μm deep and 600 μm wide). The mixing channel length was 1, 2, and 3 mm, respectively. The net flow rate was varied to achieve Re (Reynolds number) numbers in the range between 500 and 1100. The resultant product suspension was then separated and analysed with high-performance liquid chromatography (HPLC) and compared to their reference standard.

The authors reported frequent blocking of the mixers after 2–3 min of operation, with that blocking occurring predominantly at low flow rates and in mixers with a long outlet channel. The authors state that experiments with high flow rates (pump limits) could be finished without blocking and also suggested, to lower the risk of channel blockages, that the length of the mixing channel could be reduced to the minimal value necessary for good mixing. The authors also indicated that full conversion to pigment CLA1433 was not accomplished, but that the batch production process also suffered from poor conversion requiring nearly a day, and the use of additives, to reach 97% conversion [29]. Furthermore, the continuous micromixer process had a much shorter reaction time and was not optimized in terms of pH or use of additives and hence further improvements may have been possible with additional investigations.

## 2010-2016

**Fig. 6:**
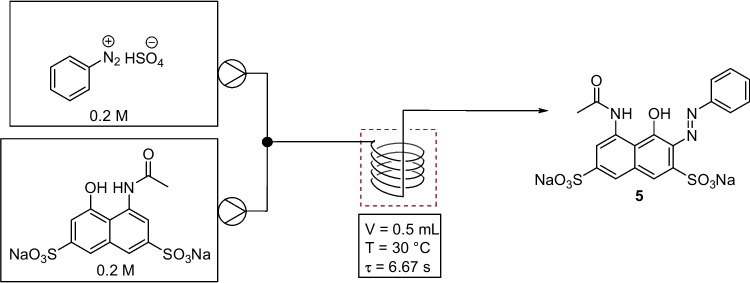
Optimised continuous flow synthesis of C.I. Acid Red 1 [30]. Diazo reagent aq. (0.2 M) flowed at a rate of 0.075 m/s and coupling reagent aq. (0.2 M) flowed at a rate of 0.075 m/s

**Fig. 7:**
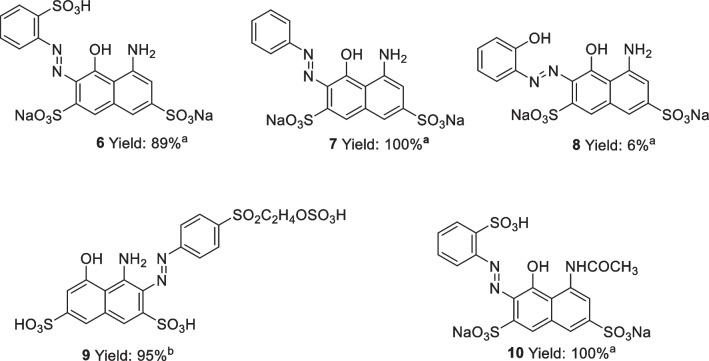
Azo dyes synthesised by Yu et al*.* ^a^ pH 8. ^b^ pH 3 [30]

In 2012, Yu et al*.* reported the continuous flow synthesis of azo dye C.I. Acid Red 1 (compound 5), Fig. 6 [30]. A simple flow system was employed using a self-made PTFE-microreactor and two single-channel micro-injection pumps. Both the diazonium and coupling starting materials were made in a batch process. The effects of flow rate, residence time, tube diameter, and reaction temperature were investigated for the azo coupling step, with optimum reaction conditions of internal tube diameter: 0.8 mm; residence time: 6.67 s; flow rate: 0.15 m/s; and temperature: 30 °C giving the product dye in a 97 % yield. A single variable was changed at a time, with residence time and temperature showing little to no effect on product yield. Flow rate did impact on product yield with rates higher or lower than 0.15 m/s resulting in a decreased yield. It was suggested that poor mixing at the higher flow rates could be responsible for the reduction in yield. One might expect improved mixing at faster flow rates, but perhaps in this case the reduced residence time (as volume fixed but flow rate increased) may have resulted in incomplete mixing in the shorter time frame and hence incomplete reaction and reduced yields. The internal tube diameter seemed to have a slight effect on yield, reaching a maximum and then decreasing again with increasing internal tube diameter. This optimised process was then applied to dyes 6-10, Fig. 7 [30].

In April 2015, Feng et al*.* reported the generation of two simple azo dyes in an undergraduate laboratory activity using a student-fabricated microreactor [31]. The microreactor was made from degassed poly(dimethylsilane) (PDMS) and a student generated polystyrene thermoplastic mould. Inlet and outlet holes were created using a biopsy punch with sixteen gauge × 1.5 in. needles attached to the tubing using a 5 min epoxy. All reagents were injected into the microfluidic device using a syringe pump (Chemyx Fusion 100). Students were encouraged to make multiple chips with varying dimensions, altering the individual residence times depending on the flow rate used.

**Fig. 8:**
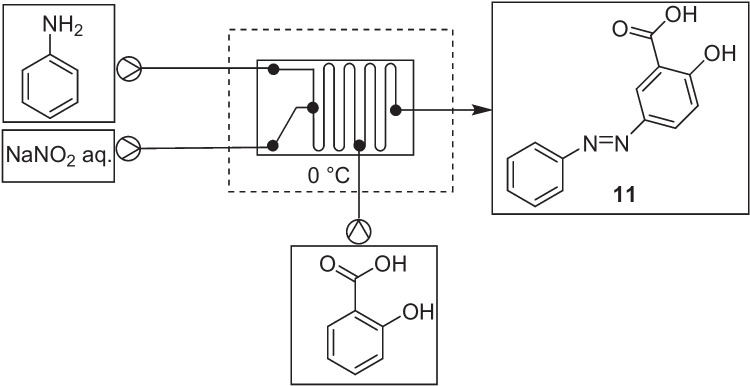
Feng et al*.*’s continuous flow synthesis of azo 11 [31].The flow conditions used were, solution A (aniline aq.), solution B (NaNO_2_ aq.) and solution C (salicylic acid aq.) were flowed at 0.09 mL/min each and the reactor chip placed on ice. Flow rates were given, however, no information on reactor volume or concentration were provided

A three pump system was used where by the diazonium salt was first generated in flow from aniline (0.09 mL/ min) and sodium nitrite (0.09 mL/ min) aqueous solution and a subsequent azo coupling with either a phenol or salicylic acid aqueous solution (0.09 mL/ min), Fig. 8 [31]. The resulting dye reaction mixture was acidified until the product precipitate and collected via filtration, with subsequent characterized using FTIR spectroscopy and comparison with commercial standards.

**Fig. 9:**
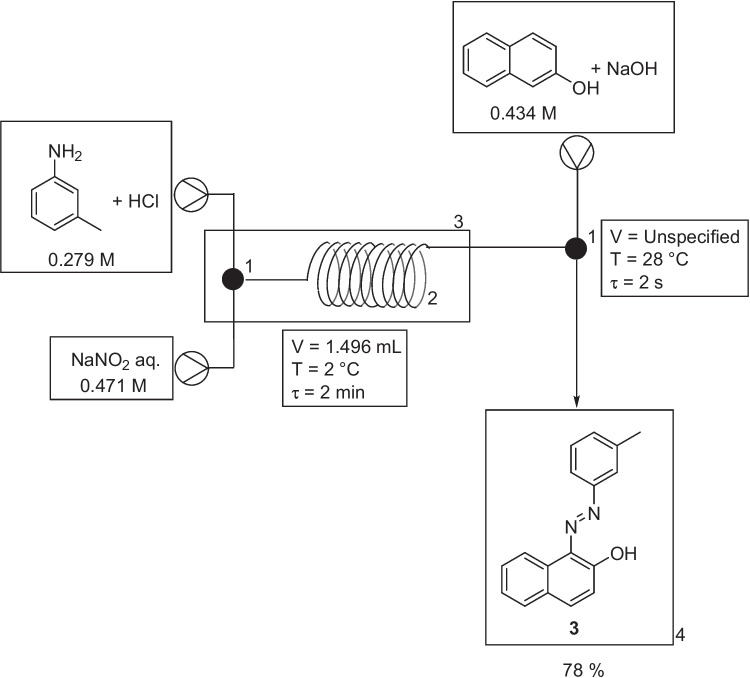
Experimental set-up used for azo coupling reaction. 1- micromixer, 2- tubular coiled reactor, 3- constant temperature baths, 4- product collected under stirring [32]. *m*-Toluidine aq. (0.279 M) plus HCl (2.23 M) flowed at a rate of 0.374 mL/min, NaNO_2_ aq. (0.471 M) flowed at a rate of 0.374 mL/min and β-naphthol aq. (0.434 M) plus NaOH (3.37 M), flow rate not provided . Volume of azo coupling micromixer not provided

In October 2015, Ranade et al*.* carried out a case study on a multi-phase, multi-step synthesis of an azo dye, (*E*)-1-(m-tolyldiazenyl)naphthalen-2-ol, Fig. 9 [32]. The typical experimental set-up used a tubular reactor for the first step and a T-mixer as a reactor for the second step. The first step was a diazotization reaction where two solutions, one of *m*-toluidine aq. (0.279 M) plus HCl (2.23 M) and a second of NaNO_2_ aq. (0.471 M), were passed continuously through a T-micromixer (1.38 mm i.d.) followed by a 1 m long SS316 tubular reactor (1.38 mm i.d. and 1.58 mm o.d.). Temperature of the reaction was controlled by immersing the tubular reactor in a thermostat-controlled water bath. The azo coupling reaction, following diazotization, employed a third solution of β-naphthol aq. (0.434 M) plus NaOH (3.47 M), the azo coupling reaction itself took place in the T-mixer (length ~5 mm, residence time ~2 s) at a constant temperature of 28 °C. The azo product was collected at the outlet in a beaker and an isolated yield of the precipitate obtained, with subsequent characterization by UV–visible spectrophotometry. Synchronization of peristaltic pumps was used to enhance pulsation effects to help achieve more effective mixing in a shorter time. Different residence times of 60 s and 120 s had little effect on yield for the diazotization reaction, but increasing temperature resulted in a reduced yield with a decrease from 98% at 0 °C to 89.1% at 30 °C for the diazonium salt. It was suggested that the unstable nature of diazonium salts led to rapid decomposition at the higher temperatures.

The authors initially conducted both steps in tubular reactors but faced blockages in the second reactor (azo coupling step). The authors indicated that increasing the residence time by moving to a larger tubular reactor was not an option, as this would result in the deposition of the diazonium salt and blocking of the channel (eventually blocking the channel or creating very high back pressure). This caused the authors to carry out the azo coupling reaction in the T-micromixer without attachment to a tubular reactor, as previously mentioned. The issue the authors encountered was that the azo coupling reaction was not complete on collection after using a residence time of 2 s (an azo dye yield of 68% was obtained). To address this issue and enhance the yield, the authors redirected the reaction mixture into a Continuous Stirred-Tank Reactor (CSTR). This modification aimed to achieve complete azo coupling while minimizing the potential for blockages in the micromixer. Consequently, the azo dye yield increased to 78%.

The authors conducted a series of experiments, where both steps were conducted in batch, both steps in continuous mode, or a combination of batch and continuous modes. For a fully batch process (10 min reaction time for step one, 30 min reaction time for step two) an azo dye yield of 98% was obtained. Batch (step one) to flow (step two), and flow (step one) to batch (step two) experiments gave yields of 62 % and 97 % respectively, and suggested that the azo dye yield was being impacted by the short residence time (2 s) of the continuous azo coupling step and therefore further supported the idea of using a continuous flow platform with the incorporation of CSTRs.

**Fig. 10:**
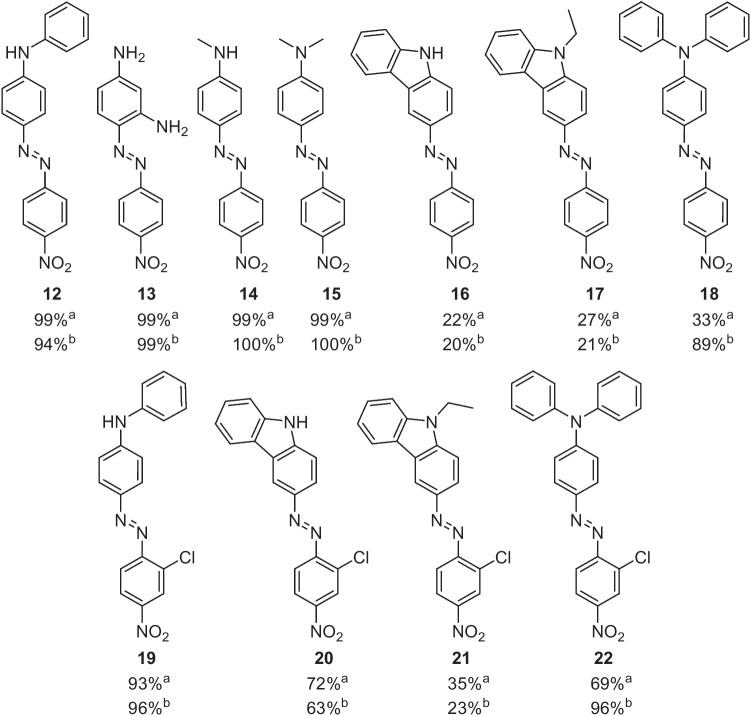
Azos synthesised by Akwi and Watts, showing conversion % for a microreactor set-up^a^ and a batch process^b^ [33]

**Fig. 11:**
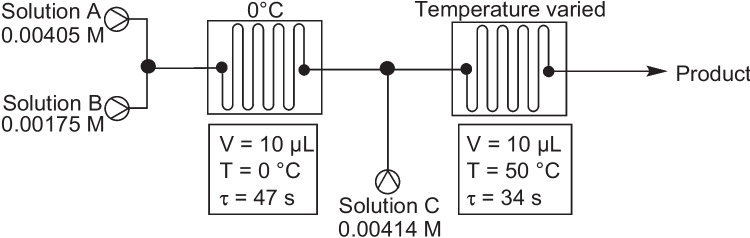
Schematic representation of the microreactor setup. Solution A- amine (4.05 mM). Solution B- sodium nitrite aq. (17.5 mM). Solution C- hydrophobic coupler (4.14 mM) and PTC catalyst [33]. The optimal flow conditions were, solution A was flowed at 9 µL/min, solution B was flowed at 3.7 µL/min and the coupler was flowed at 5.1 µL/min

In 2016, Akwi and Watts reported the use of continuous flow, microreactors, and phase transfer catalysis (PTC) in the generation azo compounds, Fig. 10 [33]. Here they utilized a phase transfer catalyst in a liquid– liquid Taylor droplet flow reaction system for the *in-situ* reactive quench of diazonium salts. The authors suggested that the plugs or slugs formed in this type of two-phase flow systems improve the mixing and mass transfer of reactants involved due to added internal circulation within these fluidic formations. In other words, these plugs or slugs act as mini microreactors existing in a microchannel of the microreactor. The continuous flow microreactor set up, Fig. 11, used three (1 mL) SGE glass syringes and syringe pumps to deliver the three-reactant solutions A (amine and HCl, water/acetonitrile, 4.05 mM), B (sodium nitrite aq., 17.5 mM), and C (hydrophobic coupler and PTC, chloroform, 4.14 mM). Two T-mixers (Chemtrix T-mixer 3023; reactor volume: 10 μL, width channel size: 300 μm, depth channel: 60 μm, and quench volume: 1.5 μL) joined by PEEK tubing (ID 125 μm) and employed temperature controller units. The PTC used was sodium dioctyl sulfosuccinate.

Several reaction parameters were explored. The number of PTC equivalents, with respect to the diazotizable amine, was studied and the conversion to the azo product (measured by HPLC) gradually increased between 0.01 and 0.2 eq. of PTC. There appeared to be no visible increase in the conversion beyond 0.2 eq. of PTC up to 0.5 eq. The flow rate of the coupling solution (C) was also studied with the predicted drop in conversion at higher flow rates (> 8 μL/min observed). The preferred flow rate of azo-coupler solution was in the range of 2–8 μL/min. With regard to the flow rate of the sodium nitrite solution (B), a flow rate in the range of 2–5 μL/min was preferred. Variation of the coupling reaction temperature was also predicted to have an effect on conversion (note the diazotization step was kept at 0 °C), with higher temperatures expected to reduce the surface tension between the two phases and facilitate the transfer of reactants across the liquid interface. The final preferred conditions were *p*-nitroaniline solution A (9 µL/min), sodium nitrite solution B (3.7 µL/min), diazotization temperature 0 °C, diphenylamine coupler and PTC solution C (5.1 µL/min), azo coupling temperature 50 °C, and PTC 0.42 equivalents, which gave a 99% conversion to the final azo product (compound 12). Additional examples were completed using 2-chloro-4-nitroaniline (solution A) and triphenylamine, 9-ethylcarbazole, and carbazole as the azo couplers. The microreactor approach was compared to a PTC batch process in each case, and although similar reaction times and conversations were found for all examples the authors emphasized that the continuous flow approach also provides enhanced safety, scalability, environmental advantages, ease of work up, and collection.

**Fig. 12:**
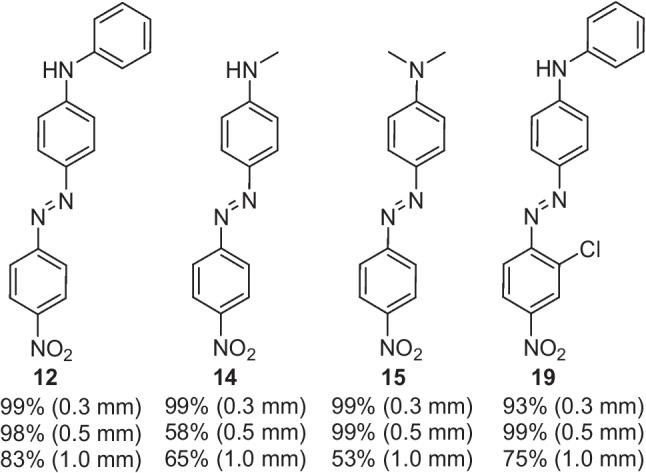
The effect of microreactor channel internal diameter, given in parenthesis (mm), on the conversion rate (%) of the four azo products synthesised [22]

Later in 2016, Akwi and Watts again reported on the use of PTC in the continuous flow synthesis of the same azo compounds (reactor channel size 0.3 mm), but this time systematically explored larger scale systems and the effect increases in channel diameter had on conversion to product [22]. Two systems were explored, one using a PTFE tube reactor with a larger channel (internal) size of 0.5 mm and the second using a Little Things Factory (LTF)-MS microreactor with a further increase in channel (internal) size to 1 mm (Fig. 12). The authors used the same experimental setup as described in their earlier PTC microreactor publication, Fig. 11 [33]. The residence time (2.4 min, controlled by changing the flow rate) in both scale-up reactors was kept constant, such that the only variable parameter was the internal diameter of the reactor channel and PTFE tubing.

The investigation of scale-up found that internal diameters of the reaction channel had an impact on the studied conversions and was most obvious for the 1.0 mm LFT-MS microreactor, which saw conversion drop significantly for all four azo compounds generated, Fig. 12. It was suggested that the increase in the internal diameter of the reactor channel had led to a decrease in surface-area-to-volume ratio, which in turn caused a limitation in the mass transfer between the continuous and dispersed phases [22]. As such, channel diameter can be considered an important parameter for the optimisation of continuous flow biphasic systems.

**Fig. 13:**
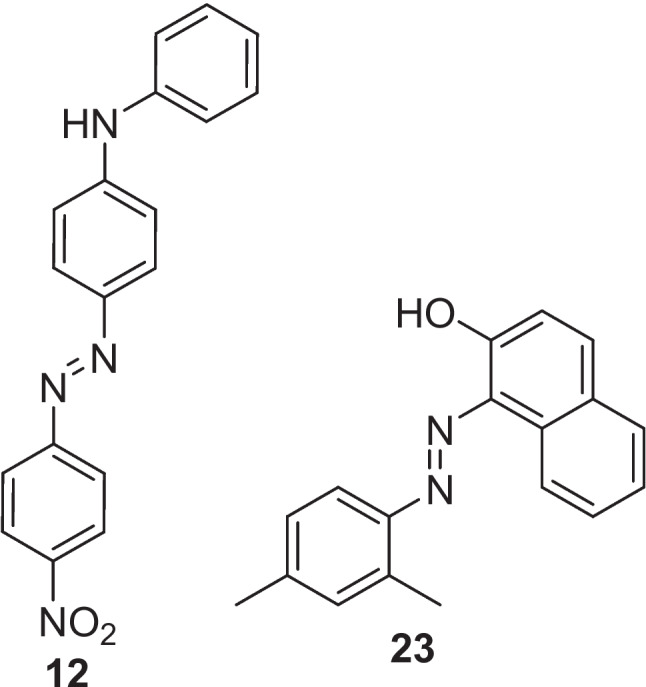
(E)-4-((4-nitrophenyl)diazenyl)-N-phenylaniline(12) and Sudan II (23) [34]

In a separate 2016 publication, Akwi et al*.* described the use of microreactors in the continuous flow synthesis of azo compounds via the *in-situ* generation of diazonium salts and their subsequent reaction with aminated or hydroxylated aromatic systems without the use of a PTC [34]. Two model azo coupling reactions were explored. One using a diazonium salt of 2,4-dimethylaniline and the hydroxylated aromatic 2-naphthol to give the azo dye Sudan II and a second reacting the diazonium salt of *p*-nitroaniline with the animated aromatic diphenylamine, Fig. 13. In both cases, changes in reaction temperature, pH, and flow rate were investigated. When investigating the azo coupling reaction of Sudan II, the authors ran 25 experiments with differing pH (5.11-10.83), flow rates (0.03-0.7 mL/min) and temperature (2-50 °C) and monitored the conversion % of each run. A quadratic model was then fitted onto the resultant calculated conversion of 2-naphthol as obtained from using reverse-phase HPLC, Table 1. The model concluded that a pH of 8.5, a flow rate of 0.03 mL/min, a residence time of 6 min 40 s, and a temperature of 25 °C would lead to an approximate conversion of 80%. A similar set of 27 experiments was ran for the azo coupling reaction of (*E*)-4-((4-nitrophenyl)diazenyl)-N-phenylaniline. The model predicted high yields of above 98% with a pH of 5.71, a flow rate range of 0.03-0.7 mL/min, a residence time of between 17 s and 6 min 40 s, and a temperature of 25 °C. For both reactions, flow rate was found to have no meaningful effect on conversion. The authors used the predicted optimised azo coupling reaction conditions for Sudan II in a two-step continuous flow synthesis involving a diazotisation reaction followed by an azo coupling reaction. Use of these conditions allowed the generation of Sudan II in a conversion of 98% along with a small library of azo compounds (79-97 % conversion). The microreactor flow set up used three (1 mL) SGE glass syringes, syringe pumps, PTFE tubing (i.d. 0.5 mm, length: 340.2 mm connecting from the first reactor plate to the second reactor plate and 380.7 mm connecting from the second reactor plate to the sample collection bottle), LTF-MS microreactor plates (reactor volume: 0.2 mL, channel size: 1 mm, geometry: 115 ×60 × 6 mm), and reactant solutions A (amine + HCl, 0.2 mL/min, 0.024 M, water, DMF), B (sodium nitrite aq., 0.07 mL/min, 0.084 M), and C (coupler + NaOH or glacial acetic acid, 0.7 mL/min, 0.049 M, water, DMF). A larger scale process was also established where it was found that a simple continuous flow set up consisting of T-mixers and PTFE tubing, with a larger i.d. of 1.5 mm for both the diazotization reaction and azo coupling reaction, provided relatively satisfactory reaction conversions (66-91 %) for eleven of the azo compounds.

**Table 1:** Optimised azo coupling flow conditions as predicted by Akwi *et al.* quadratic model [34]

Compound no.	pH	Flow rate (mL/min)	Residence time	Temperature °C	Approx. % conversion
12	5.71	0.03-0.7	17 s - 6 min 40 s	25	>98
23	8.5	0.03	6 min 40 s	25	80

## 2017-2020

**Fig. 14:**
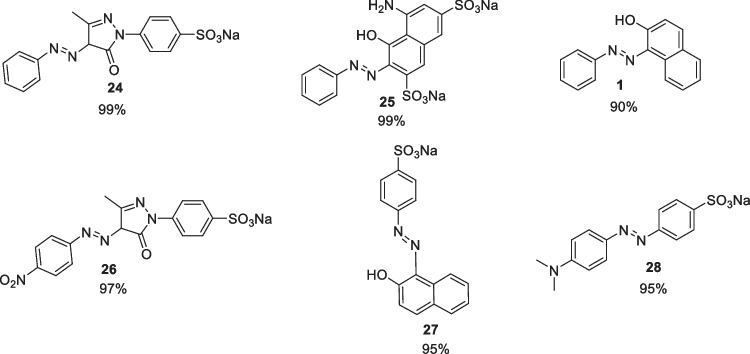
The yields of the different azo dyes under optimal conditions synthesised by Wang et al [10]

**Fig. 15:**
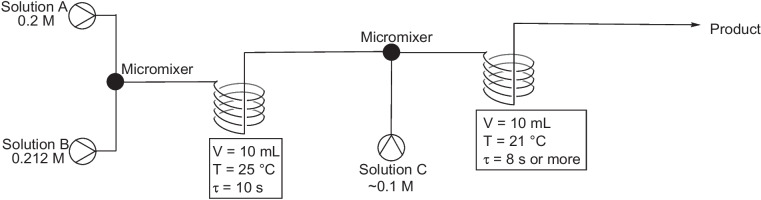
The experimental setup for continuous flow synthesis of an azo dye using a microreactor system. Optimised reaction conditions were: 1) Solution A (0.2 M): aniline + HCl in DMF:water (3:7) flowed at a rate of 30 mL/min; solution B (0.212 M): sodium nitrite in DMF:water (3:7) flowed at a rate of 30 mL/min. 2); solution C (~0.1 M): coupling components in ethanol:water (3:7) or DMF:water (3:7) , flowed at a rate of 60 mL/min, and an initial pH value of 10 [10]

In 2018, Wang et al*.* reported on the continuous flow synthesis of six azo compounds, using a microreactor, where they investigated the effect of temperature, flow rate, pH, and residence time on product yield [10]. Four model azo compounds were used to develop optimised conditions that were then applied to two commercial azo dyes, namely orange II and methyl orange, compound 27 and 28 respectively, Fig. 14. The continuous flow set up used by Wang and coworkers, Fig. 15, is a typical lay out for azo synthesis and allowed them to easily change a number of variables to find preferred reaction conditions.

For all compounds, it was shown that flow rate was the main factor affecting product yield, when flow rates increased from 10 mL/min to 60 mL/min, there was an increase in product yield due to enhanced reactant mixing. There was a significant drop off in product yield above 60 mL/min, with the authors suggesting that this was down to a short residence time within the reactor and incomplete conversion to products. Temperature changes in the range of 10 °C to 21 °C seemed to have no effect on the coupling reaction, however, temperatures above 21 °C resulted in a drastic reduction in yield. The authors put this phenomenon down to the fact that higher temperatures led to an increase in diazonium salt decomposition. The pH of the reaction was also found to affect the product with significant increases in yield as the pH increased from pH 7 to pH 10, with a maximum yield obtained at pH 10 but with a large drop in yield at pH>10. Residence time also had an impact on yield, with the different azos studies having slightly different preferred residence times, ranging from 1 s to 10 s. The residence time chosen for the synthesis of the two commercial dyes was 10 s, with the optimised reaction conditions, Fig. 15, allowing the commercial azo dyes to be generated in 95% yields [10].

**Fig. 16:**
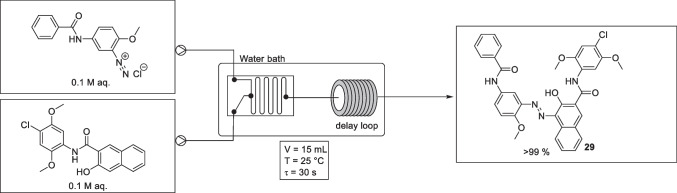
Experimental set up of Wang et al*.* C.I. PR 146 microreactor system. Optimised reactions conditions: diazonium salt solution aq. (0.1 M) flowed at a rate of 15 mL/min and coupler component aq. (0.1 M) flowed at a rate of 15 mL/min[24]

In August 2019, Wang et al*.* reported the continuous flow synthesis of a commercially valuable azo dye Pigment Red 146 (C.I PR 146), compound 29, Fig. 16 [24]. The continuous flow set up is shown in Fig. 16 and employed two stock solutions, one being a diazonium salt solution (0.1 M) in water and the second a coupler (Naphthol AS-LC, 0.1 M) solution in water, along with a stainless-steel micromixer, and a PTFE delay loop (i.d. 2 mm). Both the micromixer and the delay loop tubes were submerged in a water bath to allow control of the reaction temperature. The residence time of the reaction was controlled by changing the length of the PTFE delay loop. After collection was complete, the products were stirred for 5 mins and a resin (CAS No. 8050-09-7 [28]) solution was added into the collection tank to stop pigment crystallization. The reaction mixture was then heated to 90 °C to break up a mass of aggregated pigments and subsequently cooled to room temperature. Finally, the products were filtered by air pump filtration, washed by water, and dried for 12 h.

For reaction conditions optimisation, the microreactor was varied, along with residence time, flow rates, and temperature. The conversion of the coupling reaction was calculated using the filter paper percolation ring test, with purity being determined using a UV spectrophotometer, and particle-size distribution of the pigment studied using laser diffraction.

Variation of the microreactor included the use of a microsieve pore dispersion reactor, in which two microsieve pores were arranged side by side, and a second in which the microsieve pores were arranged back and forth in the continuous phase flow direction. A reactor with membrane dispersion was also considered. The conversion of the coupling reaction using the membrane dispersion reactor was slightly higher (99%) than that of the other mixing methods (97 % for pores side by side, 88% for pores back and forth). However, with membrane dispersion the pigment products accumulated on the surface of the membrane, clogging the micromixer, and hence was not suitable for a long-time continuous operation. The microsieve pore dispersion approach, with pores side by side, solved the clogging problems, as the pore size used (300 μm) was 2 orders of magnitude larger than the particle size of the pigments, and afforded good mixing.

Residence time, flow rate, and temperature were then studied using the preferred micromixer set up. To study the effect of changes to residence time on the coupling reaction, the delay loops lengths were varied with the flow rates of the reactants set at 30 mL/min and the reaction temperature at 20 °C. The product solution obtained at the outlet was passed directly into a strong sulfuric acid solution (pH = 1−2) to quench the coupling reaction. The conversion of the coupling reaction increased with increasing the residence time, and a conversion of >99 % was obtained when the residence time was > 30 s.

The authors also studied the effect of temperature on pigment purity, with the flow rates set at 30 mL/min, the residence time at 30 s, while changing the temperature. High purity (>95 %) of the pigment products was obtained when the reaction temperature was 25 °C, however, when the temperature was > 25 °C, purity decreased (<80 %), mostly likely due to the decomposition of the diazonium salts. The effects of the reaction temperature on the particle size and particle-size distribution was also explored, with *d*(0.1) and *d*(0.5) were essentially unchanged between 10 °C and 50 °C, but with *d*(0.9) showed an increasing trend as the temperature increased. The particle size distributions were essentially the same at the different temperatures. However, due to possible diazonium salt decomposition at higher temperatures, it was concluded that 25 °C was the preferred temperature for the azo coupling reaction.

The flow rate was varied from 10 to 50 mL/min, and its effect on conversion, particle size and particle-size distribution studied. The residence time was set at 30 s and the reaction temperature at 25 °C. Conversion increased slightly (95 to 99%) as the flow rate increased from 10 to 30 mL/min, but was essentially unchanged at flow rates > 30 mL/min. The particle size, *d*(0.9) and *d*(0.5), generally decreased as the flow rate increased, however, *d*(0.1) remained essentially unchanged. The higher flow rate resulted in narrower particle-size distributions, with a broad particle-size distribution observed at lower flow rates. It was suggested that mixing performance was improved at higher flow rates, with less back mixing and a resulting reduction in the agglomeration of pigment particles. As such, the preferred flow rate was determined to be 30 mL/min.

The rheological and colorant properties of the C.I. PR 146 generated by the continuous process were also studied and compared favourably with the C.I. PR 146 standard. An initial scaling-up study was also conducted with the flow rate of reactants increased to 100 mL/min and the number of microsieve pores increased to four (to reduce the pressure of the system). A conversion >99% was obtained, and when operating for 30 min the two pump pressures did not exceed 0.2 Mpa.

**Fig. 17:**
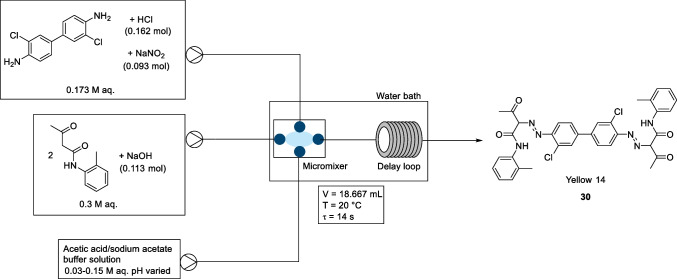
Wang et al*.* three-stream micromixing schematic for synthesis of azo dye Yellow 14. Optimised reactions conditions: diazonium salt solution (0.173 M) flowed at a rate of 10 mL/min and coupler component (0.3 M) flowed at a rate of 10 mL/min, buffer solution (0.03- 0.15 M, pH 4.35-4.60) flowed at a rate of 40 mL/min [35]

In December 2019, Wang et al*.* reported a three-stream micromixing system to synthesize azo dye Yellow 14 (compound 30), Fig. 17, and demonstrated improved performance compared to a two-stream micromixing system [35]. The two-stream process employed an N-(2-methylphenyl)-3-oxo-butanamide (AAOT) alkaline stream and a second single stream containing the diazonium salt and acetic acid. In the three-stream process, the acetic acid/sodium acetate buffer solution was pumped into the micromixer as a separate stream to the diazonium salt (0.173 M aq.) and the AAOT alkaline stream (0.3 M aq.), Fig. 17. Both the diazonium salt solution and the AAOT alkaline solution were pumped into the microchannel as dispersed phases. The three-stream microreactor was derived from previous publications by the same research group where they investigated the modelling of micromixing performance in micropore dispersion reactors [36]. The diameter of the microsieve pores was 0.3 mm, with the width and the length of the microchannel were 4 mm and 60 mm respectively. The internal diameter of the microchannel was 1 mm.

The use of the three-stream process allowed easy control of pH and enabled the group to study the effect of pH, flow rates, and temperature on the azo coupling reaction. The azo coupling reaction itself was carried out in the micromixer and then flowed into the delay loop. The reaction temperature was controlled by a water bath, and the delay loop tube had an outer diameter of 3 mm and an inner diameter of 2 mm, with a volume of 18.84 mL.

The effect different reaction conditions had on purity, particle size, and particle size distribution of the pigment products were investigated. The pH was controlled by the concentration and the volume flow rate of the acetic acid/sodium acetate buffer solution. During this experiment, the reaction conditions were as follows: the temperature was 20 °C, both the volume flow rates of the DCB diazonium salt solution and the coupling component (AAOT alkaline solution) were 20 mL/min, and the flow rate of the acetic acid/sodium acetate buffer solution was 40 mL/min. The effect of pH was studied using pHs between 3.72 and 4.54. The purity of the pigment product was shown to increase with increasing pH, with the purity reaching an optimal level, ~95%, for pH values between 4.0 to 4.3. When the pH of the product solution increased above 4.3, the purity trended downwards. It was also found that with increasing pH, the particle size decreased, and a narrower particle distribution was obtained. The authors suggested that the coupling component, (N-(2-methylphenyl)-3-oxo-butanamide), precipitates easily in the acid solution, i.e. when the pH of the azo coupling reaction is lower than 4.0, and that the pigment products will coat the coupling component particles. This causes the azo coupling reaction to be blocked, low purity, larger particle size, and a broad particle size distribution. The authors also suggest that the diazonium salt is unstable at pH >4.3, resulting in a decrease in pigment purity. However, a slight increase in particle size was observed with a pH higher than 4.0. The authors concluded that in order to obtain pigment products with high purity and narrow particle size distribution maintain, the pH of the product solution should be between 3.97 and 4.28, and this meant having the buffer solution pH in the range of 4.35 to 4.60.

To study the effect of flow rate and reaction performance, the flow rates of the DCB diazonium salt solution and the coupling component were varied, using increments of 5, from 10 mL/min to 25 mL/min. The flow rate of the acetic acid/sodium acetate buffer solution was varied, using increments of 10, from 20 mL/min to 50 mL/min. During the flow rate study, the temperature was set at 20 °C and the pH of the acetic acid/sodium acetate buffer solution at 4.60. The purity of the pigment product did increase with increasing volume flow rates, with highest purity obtained with a diazonium salt solution flow rate of 20 mL/min. Furthermore, the particle size distribution became narrower, and the particle size decreased with increasing flow rate. The authors suggested that mixing effects improved with the increase in flow rate, which in turn increased the rate of azo coupling reaction and reduced the agglomeration of the pigment particles.

Changes in reaction temperature was also considered, and experiments conducted from 10 to 30 °C, with increments of 5. The flow rates of the DCB diazonium salt solution and the coupling component were set at 20 mL/min and that of the acetic acid/sodium acetate buffer solution at 40 mL/min. The pH of the acetic acid/sodium acetate buffer solution was 4.40. As temperature increased, there was a slight downward trend in product purity, with particle size distribution showing an increasing trend when the reaction temperature went above 25 °C.

The final optimised conditions for the three-stream system were diazonium salt solution and the coupling component flow rates of 20 mL/min, a buffer solution flow rate of 40 mL/min, buffer solution pH between 4.35 and 4.60, with the reaction temperature set at 20 °C. Compared with the two-steam system previously tested by the group, the use of the three-stream process allowed the authors good control of reaction pH and after optimisation for pH, temperature and flow rates, the pigment product could be obtained in high purity with good chromatic light profile. In comparison to the two stream micromixing process the purity of the three-stream micromixing process increased from 86.4 % to 94.5 % and overall, it was suggested that the three-stream micromixing process was an improvement over the two-stream system.

## 2021-2023

**Fig. 18:**
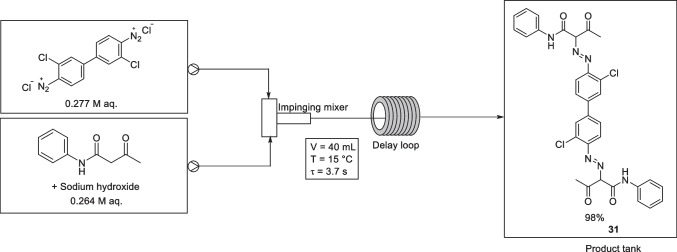
Experimental setup of Shi et al*.* C.I Pigment Yellow 12 using a high-flux continuous system. The optimised conditions were: diazonium solution aq. (0.277 M) flowed at a rate of 20 L/h, coupler solution aq. (0.264 M) flowed at a rate of 20 L/h (total residence time of 3.7 s), an acetoacetaniline temperature of 15 °C with a pH of 11.2, and a LpH of 6.0 [21]

In 2021, Shi et al*.* reported the continuous flow synthesis of C.I. Pigment Yellow 12 (PY12, compound 31), Fig. 18, using an impinging mixer and high flow rates [21]. The experimental setup employed two diaphragm metering pumps, made of polytetrafluoroethylene, to pump two reactants (A and B) into an impinging mixer. A delay loop reactor with an outer diameter of 8 mm, an inner diameter of 6 mm, and a length of 1 m was connected to the impinging mixer outlet. The products were collected in a tank at the exit of the experimental setup. The impinging mixer consists of an impinging tube and a flow barrier. The inner diameters of the mixer inlets, outlet, and impinging tube were 4, 9, and 22 mm, respectively.

The coupling component, acetoacetanilide, was pumped into the mixer as a clear alkaline (NaOH) aqueous solution with a pH of 11.2. A separate clear diazonium solution was also pumped into the mixer. Adjustment of the pH of the acetoacetaniline solution, by the addition of acetic acid and sodium acetate to the stock solution, allowed the local pH of the azo coupling reaction to be regulated. The local pH of the azo coupling reaction was determined using a pH electrode in the impinging mixer. The optimum pH for the azo coupling reaction was suggested to be in the range of 4.5-6, and an acetoacetaniline solution pH of 11.2 resulted in an azo coupling reaction pH of ~5.2.

Fast heat transfer resulted in no accumulation of heat in the mixer and the delay loop reactor. When the temperature of the acetoacetaniline solution was set to 5, 10, or 15 °C, the PY12 yields were reported to be above 98%, without significant differences between them. However, with further temperature increases to 20 and 25 °C, the yield decreases to 96.6 % and 93.4 %, respectively. The authors suggested that the reduction in yield could be due to the instability of diazonium salts at higher temperatures.

The PY12 yield increased obviously from 92.3 to 98.5% as the flow rate was increased from 20 to 40 L/h, with the improvement performance assigned to enhanced mixing efficiency. Further increases in flow rate resulted in a decreased yield and was attributed to the short residence time and an incomplete reaction.

The final optimized process employed an acetoacetaniline solution with a pH of 11.2 and temperature of 15 °C, a local pH (LpH) of 6.0, and a flow rate of 40 L/h. These conditions furnished PY12 in a yield of 98%, with a smaller particle size and better dispersity than the commercial batch products, leading to a higher transparency as well as a higher tinting strength. Furthermore, in comparison with the batch process, the continuous flow process in this work was reported to display decreased energy consumption, a reduction in the amount of wastewater, as well as the chemical oxygen demand (COD) of the resulting wastewater.

**Fig. 19:**
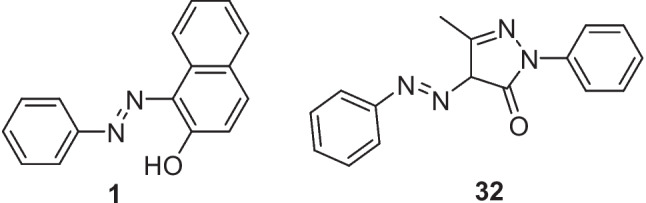
Sudan-I (1) and Solvent Yellow 16 (32) synthesised using a bubble column reactor in a continuous flow fashion by Shukla et al [37]

**Fig. 20:**
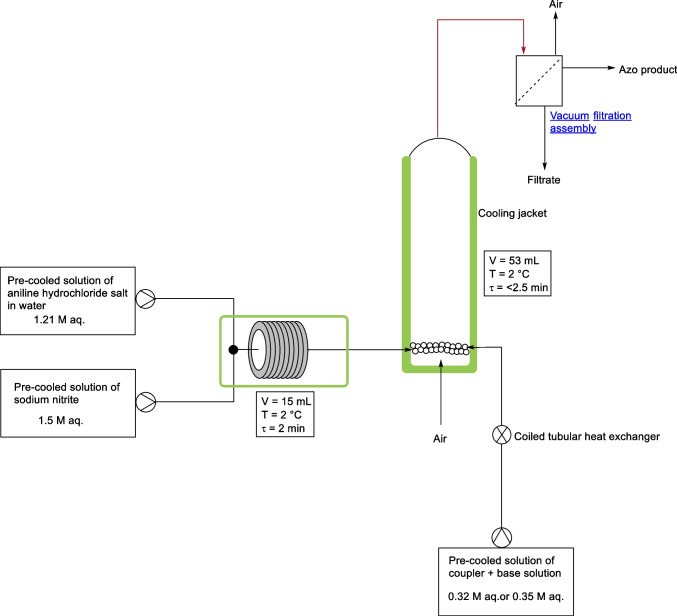
Continuous flow process diagram for the synthesis of azo dyes **1** and **32** as reported by Shukla et al. Aniline hydrochloride salt aq. (1.21 M) flowed at a rate of 3.75 mL/min, NaNO_2_ aq. (1.5 M) flowed at a rate of 3.75 mL/min and coupler aq. (0.32/0.35 M) flowed at a rate of 13.7 mL/min [37]

In 2021, Shukla et al*.* employed a bubble column reactor in a continuous flow system for the synthesis of azo dyes [37]. Their experiments successfully produced Sudan-I (compound 1) and Solvent Yellow 16 (compound 32) azo dyes with high yields of 92 % and 96 % respectively, Fig. 19. The experimental procedure for the continuous process of the azo dyes used three solutions, a solution of aniline and 35% hydrochloric acid giving a feed solution of 1.21 M, a solution of sodium nitrite (1.5 M) and a solution of the coupling substrate (β-naphthol for Sudan-I or 3-methyl-1-phenyl-1H-pyrazol-5(4H)-one for Solvent Yellow 16) dissolved in an aqueous alkaline solution of sodium hydroxide or sodium carbonate to give a 0.35 M or 0.32 M final feed solution. The feed solutions were precooled to <5 °C using an ice bath and jacketed tubular heat exchanger. The continuous flow schematic setup of Shukla et al*.* experiment is shown in Fig. 20. Peristaltic pumps were used along with a jacketed tubular reactor for the diazotization reaction (residence time of 2 min). The resulting diazonium salt was subsequently reacted with the coupling substrate in a second reactor, a jacketed bubble column reactor, with a residence time less than 2.5 min. A sintered disk was used as a sparger and a peristaltic pump was used for passing air, with the product separated using continuous vacuum filtration. Both reactors were maintained at 2 °C using a thermostat. The bubble column reactor consisted of a horizontal outlet which was connected to a pump to remove the slurry product. Clogging in the horizontal outlet tube of the bubble column reactor was observed, so to overcome this issue, Shukla et al*.* modified the reactor outlet by giving it a U-shaped overhead flow outlet at the top of the vessel and closed the horizontal outlet.

The authors also investigated the sustainability of their process and reported that compared to the corresponding batch process it reduced costs by a factor of 4.69, lowered the plant footprint, by a factor of 2.5-4.5 (depending on different refilling strategies for feed storage tanks) and that the continuous process required 39.51-41.98% less water than the typical industrial batch process. The authors noted that on scale up of this process, they were only able to perform the flow for 27 minutes (more than 10 residence times) before they encountered some clogging issues. However, the yield and productivity for Sudan-I was experimentally found to be 92 ± 3.5 % and 1.68 ± 0.13 kg/day respectively, with that of Solvent Yellow 16 azo dye determined to be 96 ± 2.2 % and 1.92 ± 0.04 kg/day respectively.

**Fig. 21:**
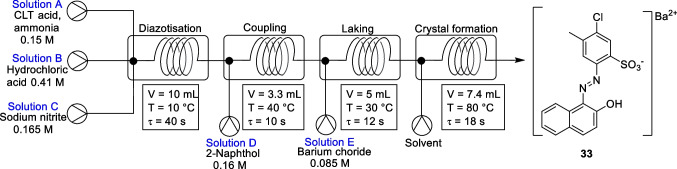
Continuous flow set-up for the synthesis of P.R.53:1. Solution A: 2-amino-4-methyl-5-chlorobenzene sulfonic acid (CLT), ammonia aq. (0.15 M). Solution B: HCl aq. (0.41 M). Solution C: sodium nitrite aq. (0.165 M). Solution D: 2-naphthol/sodium hydroxide aq. (0.16 M). Solution E: barium chloride aq. (0.085 M). The flow rate of each of the pumps were set to 5 mL/min [38]

In 2023, Mao et al*.* developed a four-step continuous flow procedure to synthesise Pigment Red 53 (compound 33) [38]. This process avoided the problems of conveying highly insoluble reaction intermediates by removing intermediate operating steps. In their work, they developed a multi-step continuous integrated production system for the diazotization-coupling-laking-crystal transition process of azo lake pigment generation, Fig. 21 (note, a lake pigment is one that is made by precipitating the dye with an inert binder such as a metallic salt). After the optimization of process parameters, the overall yield of the diazotization-coupling-laking-crystal transition process for the synthesis of Pigment Red 53:1 reached 97.1% in the total residence time of 80 s.

During an initial run of the multi-step continuous system, it was confirmed by HPLC that all the dye had been converted to laked pigments in the final laking reaction. Therefore, the authors concluded that only optimisation of the preceding diazotisation and coupling reactions were required to increase the overall yield. The effects of diazotisation time (20, 40, 60 s) and temperature (5, 10, 15, 20 °C) on the total yield were investigated. The coupling reaction conditions were fixed at residence time = 30 s, T = 40 °C and pH 8. The highest overall yield was reached at 92% when the diazotisation reactions residence time was set to 60 s with a temperature of 5 °C. The yields did not improve with an increase in temperature, and it was suggested that this was due to the thermal instability of the diazonium salt. However, to strike a balance between the yield and energy consumption, the diazotisation reaction was carried out at a residence time of 40s with a temperature of 10 °C, giving a yield of 91%.

The authors explored the influence of reaction time, temperature, and pH on the coupling step by using an orthogonal experiment with the three factors at three levels in its design. The extent of influence that each of the factors had was shown to be greatest for pH and temperature, with residence time having little effect on the yield. After the orthogonal test, the optimal conditions for the azo coupling step were determined to be, by calculation, residence time = 10 s, T = 40 °C and pH 7.5. The overall process yield was 93.5% under these optimal conditions and the stability of the result was verified by three repeated experiments.

This diazotization-coupling-laking-crystal transition process were carried out in both a continuous flow and batch fashion to verify the advantage of continuous flow. The purity of P.R.53:1 synthesized in continuous flow was found to be 98.2%, whereas the pigment product synthesized in batch had a purity of 97.1% and the median diameter of pigment particles decreased from 14 μm (batch) to 1.9 μm (continuous flow). The authors suggested that the improvement in purity is due to the superior mass and heat transfer characteristics of the continuous flow system.

## Conclusion

Continuous flow is an attractive approach for the development of safe and versatile synthetic processes, and in the case of azo and azo dye synthesis has the potential to improve scale up, environmental footprint, product purity, and colour properties. Scale up can also be achieved through numbering up of microreactors. With the exception of one paper (Shukla et al., molar range [37]), the reagent concentrations fell within the micro/milli molar range. This is likely attributed to challenges relating to solubility that can arise when working with highly concentrated solutions in the molar range. Shukla et al. faced clogging issues when working on a molar scale, with the horizontal outlet tube of the bubble column reactor becoming obstructed within a few minutes of reaching steady state. The authors adapted the reactor outlet by giving it a U-shaped overhead flow outlet at the top of the vessel and closed the horizontal outlet allowing them to perform the flow for 27 minutes (more than 10 residence times) before blockages was encountered. While the authors successfully addressed the initial blockage issues, this case underscores the persistent challenges associated with scaling up the continuous flow process.

The use of microreactor technology has demonstrated significant suitability for synthesizing azo compounds in pharmaceutical and research laboratories. Microreactors offer distinct advantages over conventional batch processes, primarily due to their high surface-area-to-volume ratio and efficient mass/heat transfer. These benefits are particularly pronounced on the micro scale compared to larger flow systems. However, a major challenge associated with microreactor technology in azo synthesis is the potential for clogging or blockages in the reactor channel. Typically, authors have opted to maintain low reagent concentrations and employed solvent systems with good solubility for their reagents/products. Solvent selection is an important consideration as azo compounds can exhibit low solubility and tend to precipitate out of reaction mixtures. Many of azo flow studies have employed a DMF-water mixture to solubilize starting reagents/products, Table 2. Although increased flow rates have proven effective in overcoming blockage problems, this approach often comes at the expense of reduced yields owing to the shortened residence times.

**Table 2:** Overview table on compatible solvents, hazards, risk mitigation for azo generation using continuous flow

Reference	Compatible Solvents ^a^	Hazards	Risk Mitigation
10, 25, 34	DMF-Water	DMF	Micro scale.
		Diazonium stability	Generated in flow, micro scale.
		Exothermic	Generate in flow (good heat transfer), micro scale.
		Blocking	Choose solvent system that has good solubility, low concentrations.
25, 33	Acetonitrile-Water	Diazonium stability	Generate in flow, micro scale;
		Exothermic	In flow (good heat transfer), micro scale
		Blocking	Choose solvent system that has good solubility, low concentrations, increase flow rate
21, 24, 28 ,30, 31, 32, 36, 37, 38.	Water	Diazonium stability	Generate in flow, micro scale.
		Exothermic	Generate in flow (good heat transfer), micro scale.
		Blocking	Choose solvent system that has good solubility, low concentrations, increase flow rates, use CSTRs. Increase flow rate

^a^ Solubility of individual azos vary and can require individual solvent screens to identify suitable solvents for dissolution

Key reaction variables to consider when developing a continuous flow synthesis of azo compounds include pH, flow rate, mixing, residence time, temperature, reactor, channel size, and the reactivity of the diazonium salt and azo coupler. The impact of changes to these variables on conversion, product purity, and product properties is not uniform and the effect they have can depend on the particular azo target.

pH was found to be a significant factor when a phenol coupler was used, with some examples showing basic pH ranges that increased conversion, decreased particle size, and narrowed particle size distribution. Approaches used to the control pH depended on the experimental set up and reactions involved, but included the use of stock reactants buffered to a specific pH and the use of a separate buffer reservoir that is mixed with the reactant solutions under continuous flow conditions.

Flow rate and mixing also impacted reaction performance with some reports detailing higher flow rates that lead to an improvement in mixing and consequently higher conversions. In some cases, improved mixing was also shown to improve glossiness and transparency of the desired azo dye, while others concluded that higher flow rates led to a narrower particle size distribution.

Optimal or preferred residence times varied for different azo couplings, and in some reports were not clearly separated from flow rate effects. Long residence times were not normally required, and residence times in the range of 1-180 seconds typically gave good conversions. Reaction temperature is an important consideration, with its effect most often attributed to the instability of the diazonium salt at higher temperatures. In some reports, increases in temperature was also shown to have a slight downward trend in product purity and a broadening of particle size distribution.

Changes in reactor type and channel size also featured in several reports. Some authors suggested that a smaller internal diameter of the channels or reactor tubing led to higher conversions. This was attributed to the improvement in mixing that occurred in the smaller tube volumes.

Colour properties are the distinctive characteristics defining how colours interact with light. The primary colour properties include hue, saturation, and brightness. Modifying reaction parameters throughout the synthetic process can enable the improvement of the sought-after colour properties and therefore reduce waste and improve cost effectiveness. The improved colour properties observed in the synthesis of azo dyes under continuous flow conditions resulted from the enhanced conversion of starting materials into the desired azo compounds, minimizing the formation of by-products. Some reports focus on dye/colour properties of a “product mixture” which can contain by-products in addition to the target azo compound. Improvements, such as by-product minimization, can be attributed to the improved mixing and heat/mass transfer observed in continuous flow conditions compared to traditional batch conditions. Colour properties are affected when using conventional synthesis, particularly upon scale up, due to the increased reaction volume and loss of mixing quality. The reactivity/stability of the particular diazonium salt and azo coupler used has a significant impact the outcome of the azo coupling reaction. For example, the stability of the diazonium salt and its rate of decomposition compared to the azo coupling rate of reaction can have significant consequences for conversion to product. Also, phenolic and amino couplers have significantly different reactivities and are impacted differently by pH.

## Perspectives

There are no reports of successful large scale continuous flow processes for azo generation, although the industry level generation of azos does occur via batch processes. Difficulties relating to poor mixing, split-flow, and blocking have hampered progress in some cases and future studies focused on scale up would need to consider approaches for improved mixing in combination with larger reactor/channel sizes. The further exploration of elevated temperatures is also warranted. Reports of continuous flow processes involving diazonium salts and other thermally unstable intermediates are common, where higher flow rates and shorter residence times can enable the successful completion of sensitive reactions at elevated temperatures. The use of such an approach for azo formation would depend on the individual diazonium species in question and the rate of the diazonium decomposition versus the rate of the azo coupling reaction. The reagent concentrations primarily fell within the micro/milli molar range. This is likely attributed to challenges related to solubility that arise when working with highly concentrated solutions in the molar range. Future investigations may need to emphasize strategies for overcoming these challenges in order to optimize production performance.

Many of the studies to date have either focused on conversion and not isolated azo yield, or on the yield of a product dye mixture as opposed to accurately determining the yield of a single pure azo product. If the ultimate goal is to develop the most efficient process in terms of atom economy, environmental footprint, waste streams, yield etc., then it is important that future work on azo generation in flow targets isolated yield and colour properties of pure azo compound and not a dye product mixture that can include unknown entities.

The continuous flow synthesis of each target azo product will typically need to follow its own optimisation process due to the variation in diazonium stability and coupler reactivity. The use of design of experiments (DoE) and/or other statistical methods could significantly improve the efficiency of the optimisation study and reduce experimentation. Some initial work in this direction was reported Akwi et al*.* where they have reported the use of a statistical model to predict optimised reaction conditions, although they did not report the experimental evaluation of the predicted conditions. The use of a continuous flow DoE type approach, coupled with inline monitoring and real time reaction optimisation, could result in more efficient and environmentally friendly methods for azo generation.

Continuous flow has considerable potential to improve the synthesis and generation of valuable azo compounds and dyes and significant progress has been made. Challenges do remain however, and further work is required to establish industry relevant larger scale processes.
